# Acid Sphingomyelinase Is a Modulator of Contextual Fear

**DOI:** 10.3390/ijms23063398

**Published:** 2022-03-21

**Authors:** Iulia Zoicas, Johannes Kornhuber

**Affiliations:** Department of Psychiatry and Psychotherapy, Friedrich-Alexander University Erlangen-Nürnberg (FAU), 91054 Erlangen, Germany; johannes.kornhuber@uk-erlangen.de

**Keywords:** ASM, knock-out mice, transgenic mice, contextual fear, cued fear, fear learning, fear conditioning, fear extinction, PTSD

## Abstract

Acid sphingomyelinase (ASM) regulates a variety of physiological processes and plays an important role in emotional behavior. The role of ASM in fear-related behavior has not been investigated so far. Using transgenic mice overexpressing ASM (ASMtg) and ASM deficient mice, we studied whether ASM regulates fear learning and expression of cued and contextual fear in a classical fear conditioning paradigm, a model used to investigate specific attributes of post-traumatic stress disorder (PTSD). We show that ASM does not affect fear learning as both ASMtg and ASM deficient mice display unaltered fear conditioning when compared to wild-type littermates. However, ASM regulates the expression of contextual fear in a sex-specific manner. While ASM overexpression enhances the expression of contextual fear in both male and female mice, ASM deficiency reduces the expression of contextual fear specifically in male mice. The expression of cued fear, however, is not regulated by ASM as ASMtg and ASM deficient mice display similar tone-elicited freezing levels. This study shows that ASM modulates the expression of contextual fear but not of cued fear in a sex-specific manner and adds a novel piece of information regarding the involvement of ASM in hippocampal-dependent aversive memory.

## 1. Introduction

Acid sphingomyelinase (ASM) is a lysosomal glycoprotein that plays an important role in the sphingolipid metabolism as it catalyzes the hydrolysis of sphingomyelin to ceramide and phosphorylcholine at acidic pH [1]. Given that lysosomes are constantly recycling to the plasma membrane, ASM can also be found on the cell surface, where it binds to the outer leaflet of the plasma membrane [2,3,4]. In addition, ASM can be secreted and act at the cell surface [4]. Ceramide molecules are highly hydrophobic and spontaneously associate with each other to form small ceramide-enriched membrane domains. These small domains already serve as signaling platforms but are able to fuse and form large and tightly packed ceramide-enriched membrane domains called “platforms” [2,5]. These platforms serve to cluster, aggregate and reorganize receptor and signaling molecules [2,6,7]. Thus, the generation of ceramide by ASM dramatically alters the biophysical properties of the plasma membrane. Similarly, changes in sphingomyelin levels induced by the activity of ASM modulate the organization of membrane receptors and signal transduction [8].

ASM regulates a variety of physiological and pathophysiological processes, including apoptosis, immune responses and inflammation, and plays an important role not only in tumor development, cardiovascular and respiratory disorders but also in neurological and psychiatric disorders [9,10]. Clinical studies revealed an increased ASM activity in the peripheral blood mononuclear cells of patients with major depressive disorder (MDD) [11], which positively correlated with symptom severity and predicted the improvement of depressive symptoms during therapy [12]. Such alterations in ASM activity might involve changes in alternative splicing of the gene coding for ASM, which differed between patients with MDD and healthy controls [13,14]. Consequently, plasma ceramide levels were increased in patients with MDD and bipolar disorder [15,16]. Elevated plasma ceramide levels were also associated with increased severity of depression symptoms in patients with coronary artery disease [17]. Similarly, post-traumatic stress disorder (PTSD) was associated with elevated plasma ASM activity and ceramide levels [18]. An increased ASM activity was also described in the plasma and peripheral blood mononuclear cells of alcohol-dependent patients [19,20,21,22]. Decreased ASM activity and elevated ceramide levels were found in the cerebrospinal fluid of patients with Alzheimer’s disease [23,24]. These studies demonstrate an altered ASM/ceramide system in several neurological and psychiatric disorders.

Animal studies also described an important involvement of ASM, especially in anxiety- and depressive-like behavior, but also in alcohol consumption. Increased ASM activity and ceramide levels in the prefrontal cortex and hippocampus, as well as reduced neurogenesis typical for depression, were described in mouse models of stress-induced depression, such as chronic unpredictable mild stress and repeated administration of corticosterone [25,26,27]. A depressive-like behavior and a reduction in neurogenesis were described in transgenic mice overexpressing ASM (ASMtg) even in the absence of stress [25]. These ASMtg mice showed increased ASM activity and ceramide levels in the hippocampus [25] and displayed increased anxiety [25,28], increased alcohol consumption [29] and a facilitated establishment of the conditioned behavioral effects of alcohol and, thus, drug memories [29] despite unaltered social and non-social memory abilities [28]. Interestingly, female but not male ASMtg mice also showed increased social anxiety [28], suggesting an association between increased ASM activity and deficits in social behavior in females. Sex-specific and brain-regional effects of ASM activity on emotional behavior were also described in transgenic mice with forebrain-specific overexpression of ASM (ASMtg_fb) [30]. Male ASMtg_fb mice showed higher ASM activity in the frontal cortex, hippocampus, amygdala and lateral septum and higher ceramide levels in the hippocampus that resulted in a depressive-like phenotype, whereas female ASMtg_fb mice showed higher ASM activity in the hypothalamus and a social anxious phenotype but not a depressive-like phenotype [30].

ASM deficient mice, on the other hand, displayed an age-dependent accumulation of sphingomyelin and development of Niemann–Pick disease type A and B, depending on the residual activity of ASM [31]. Homozygous ASM deficient (ASM−/−) mice develop normally until about 12 weeks of age when ataxia and mild tremors become noticeable. The disease progresses rapidly so that neurodegeneration similar to Niemann–Pick disease develops after only a few months of age [32]. When tested before reaching 12 weeks of age, ASM−/− mice present decreased hippocampal ceramide levels, increased neurogenesis [25] and an improved neuronal regeneration after mild traumatic brain injury [33]. At the behavioral level, ASM−/− mice are less anxious, show reduced depressive-like behavior and unaltered alcohol consumption despite enhanced alcohol preference [25,34]. Heterozygous ASM deficient (ASM+/−) mice do not show neurodegeneration but display age-dependent deficits in motor function [35,36]. At the behavioral level, ASM+/− mice display increased anxiety [37], reduced depressive-like behavior [37] and unaltered alcohol consumption despite decreased alcohol preference [29]. The pharmacological inhibition of ASM activity via administration of functional inhibitors of ASM (FIASMAs) [38], such as amitriptyline and fluoxetine, reduced hippocampal ceramide concentrations, increased neurogenesis and exerted antidepressant and anxiolytic-like effects in mice exposed to chronic unpredictable mild stress [25,27] and in ASMtg mice [25] but not in ASM−/− mice [25], further supporting the critical involvement of ASM in anxiety- and depressive-like behavior.

However, no studies to date have investigated the role of ASM in conditioned fear. An involvement of ASM is likely given that PTSD was associated with elevated plasma ASM activity and ceramide levels [18]. Animal models of Pavlovian fear conditioning are used to investigate specific attributes of PTSD, such as impaired safety learning (i.e., inability to extinguish fear memories), amplified fear responses to trauma- or stress-related stimuli and fear generalization [39]. Pavlovian fear conditioning is a type of learning in which an association between a stimulus and its aversive consequences is formed. Cued fear conditioning involves the presentation of a neutral stimulus, such as a tone or a light (conditioned stimulus; CS), in association with an aversive stimulus, such as a mild electric foot shock (unconditioned stimulus; US). Through repeated CS–US associations, animals learn that the CS predicts the US, and a conditioned fear response, such as freezing [40], is elicited in the absence of the US. In contextual fear conditioning, animals learn to associate a neutral context (i.e., the environment; CS) with an aversive stimulus (US) and display fear responses in a context that predicts danger. Fear extinction is regarded as a form of new learning [41] and is defined as the attenuation of the conditioned fear response by repeated exposure to the CS without the US. Pavlovian fear conditioning offers a unique framework for translational studies as the behavioral and physiological fear responses, as well as the neural circuits underlying fear learning, fear expression and fear extinction, are highly conserved across species [42,43,44,45].

## 2. Results

**Figure 1:**
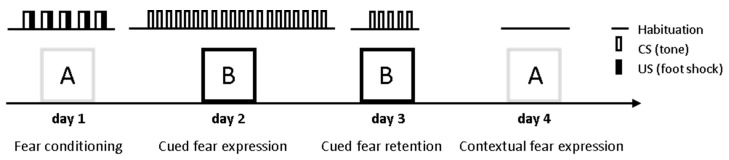
Schematic representation of the fear conditioning procedure. During fear conditioning on day 1, mice were placed in the conditioning chamber (context A) and, after a 5-min adaptation period, were exposed to five CS–US pairings. The conditioned stimulus (CS) was an 80 dB, 8 kHz, 30 s sine-wave tone, which co-terminated with a mild electric foot shock (unconditioned stimulus, US; 0.7 mA, pulsed current, 2 s). During cued fear extinction training on day 2, mice were placed in a different box (context B) and were exposed to 20 CS presentations to assess the cue-induced fear response. During cued fear extinction retention on day 3, mice were placed again in context B and were exposed to five CS presentations. During contextual fear measurement on day 4, mice were placed in the conditioning chamber (context A) and freezing was assessed as an indicator of contextual fear expression for 5 min.

In order to investigate whether ASM plays a role in conditioned fear behavior, the ASMtg, ASM+/− and ASM−/− mice were tested in a classical cued and contextual fear conditioning paradigm and compared with respective WT littermates. The male and female mice were analyzed separately to verify possible sex-specific effects. During fear conditioning on day 1, the mice were placed in the conditioning chamber (context A) and were exposed to five CS–US pairings. The CS was an 80 dB, 8 kHz, 30 s sine-wave tone that co-terminated with a mild electric foot shock (US, 0.7 mA; pulsed current, 2 s). During cued fear extinction training on day 2, the mice were placed in a different box (context B) and were exposed to 20 CS presentations to assess the cue-induced fear response. During cued fear extinction retention on day 3, the mice were placed again in context B and were exposed to five CS presentations. During contextual fear measurement on day 4, the mice were placed in the conditioning chamber (context A) and freezing was assessed as an indicator of contextual fear for 5 min (shown as 10 × 30 s time-bins) (Figure 1).

### 2.1. ASMtg Mice

**Figure 2:**
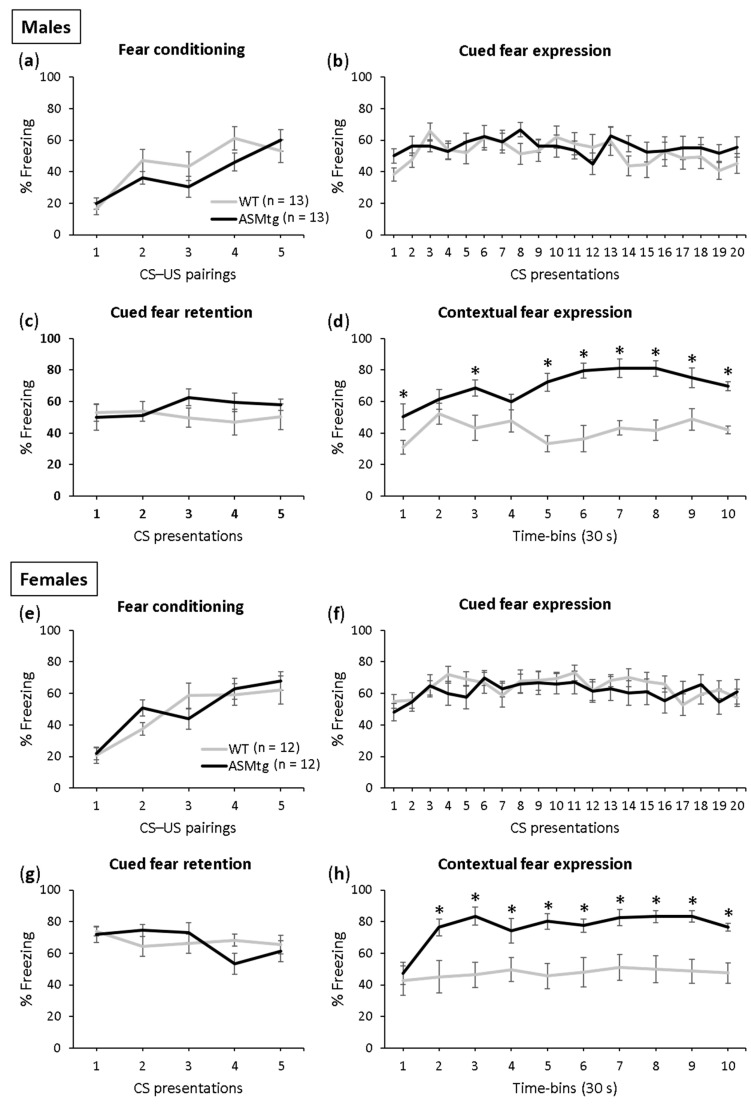
Acid sphingomyelinase (ASM) overexpression increases the expression of contextual fear in both male and female mice. (**a**,**e**) On day 1, the ASM transgenic (ASMtg) mice and wild-type littermates (WT) were fear conditioned in context A; (**b**,**f**) on day 2, the expression of cued fear (i.e., tone-elicited freezing) was assessed in context B; (**c**,**g**) on day 3, the retention of cued fear was assessed in context B; (**d**,**h**) on day 4, the expression of contextual fear was assessed in context A. Data represent the mean time of CS-elicited freezing ± SEM, and numbers in parentheses indicate group sizes. * *p* < 0.05 compared with WT mice.

Fear conditioning was successful in both the male (Figure 2a; F(4,96) = 16.000; *p* < 0.001) and female (Figure 2e; F(4,88) = 11.736; *p* < 0.001) ASMtg and WT control mice as the level of freezing increased across the CS–US pairings. There was no difference in conditioning between the ASMtg and WT controls in the male (F(1,24) = 0.963; *p* = 336) and female (F(1,22) = 0.010; *p* = 0.922) mice, which indicates comparable fear learning between the genotypes.

The male and female ASMtg and WT controls showed similar expression (males: Figure 2b; F(1,24) = 0.731; *p* = 0.401; females: Figure 2f; F(1,22) = 0.511; *p* = 0.482) and retention of cued fear (males: Figure 2c; F(1,24) = 1.025; *p* = 0.321; females: Figure 2g; F(1,22) = 0.027; *p* = 0.870), which was indicated by similar tone-elicited freezing levels during cued fear extinction training and retention on days 2 and 3, respectively. Neither the male nor female ASMtg or WT mice showed within-extinction of cued fear (males: Figure 2b; F(19,456) = 1.544; *p* = 0.067; females: Figure 2f; F(19,418) = 1.356; *p* = 0.144), as suggested by the lack of gradual decrease in the tone-elicited freezing levels during cued fear extinction training on day 2 as a result of repeated exposure to the CS without the US. This confirms previous studies using this paradigm in mice [46,47]. Similarly, neither the male nor female ASMtg or WT mice showed between-extinction of cued fear (males: F(1,48) = 0.016; *p* = 0.900; females: F(1,44) = 1.959; *p* = 0.169), as suggested by the similar tone-elicited freezing levels between cued fear extinction training on day 2 and cued fear extinction recall on day 3.

A significant genotype effect was found in the expression of contextual fear in both the male and female mice (males: Figure 2d; F(1,24) = 55.454; *p* < 0.001; females: Figure 2h; F(1,22) = 17.987; *p* < 0.001). The ASMtg mice showed increased context-elicited freezing levels compared with WT controls on day 4, suggesting that ASM overexpression enhances the expression of contextual fear in both male and female mice.

### 2.2. ASM Deficient Mice

**Figure 3:**
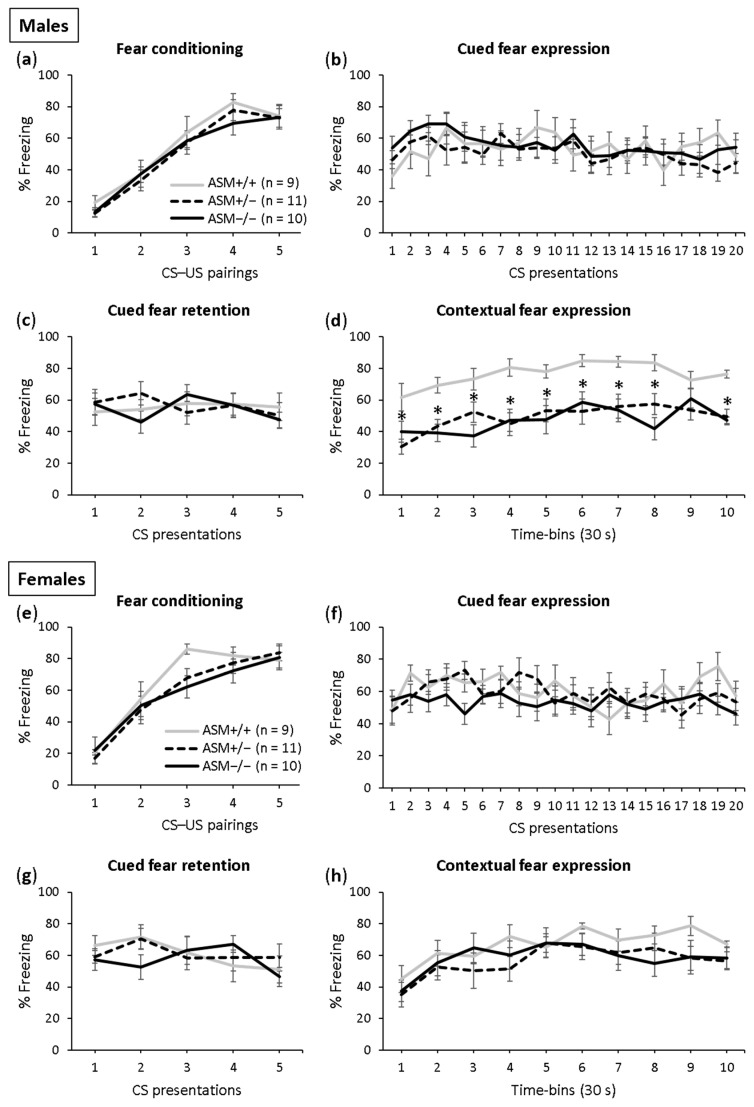
Acid sphingomyelinase (ASM) deficiency decreases the expression of contextual fear in male mice. (**a**,**e**) On day 1, the homozygous (ASM−/−) and heterozygous (ASM+/−) ASM deficient mice and wild-type littermates (ASM+/+) were fear conditioned in context A; (**b**,**f**) on day 2, the expression of cued fear (i.e., tone-elicited freezing) was assessed in context B; (**c**,**g**) on day 3, the retention of cued fear was assessed in context B; (**d**,**h**) on day 4, the expression of contextual fear was assessed in context A. Data represent the mean time of CS-elicited freezing ± SEM, and numbers in parentheses indicate group sizes. * *p* < 0.05 compared with ASM+/+ mice.

Fear conditioning was successful in both the male (Figure 3a; F(4,108) = 76.112; *p* < 0.001) and female (Figure 3e; F(4,108) = 52.106; *p* < 0.001) ASM−/−, ASM +/− and ASM+/+ (WT) control mice as the level of freezing increased across the CS–US pairings. There was no difference in conditioning between the genotypes in the male (F(2,27) = 0.376; *p* = 690) and female (F(2,27) = 0.722; *p* = 0.495) mice, which indicates comparable fear learning.

The male and female ASM−/−, ASM +/− and ASM+/+ control mice showed similar expression (males: Figure 3b; F(2,27) = 0.297; *p* = 0.745; females: Figure 3f; F(2,27) = 0.705; *p* = 0.503) and retention of cued fear (males: Figure 3c; F(2,27) = 0.062; *p* = 0.940; females: Figure 3g; F(2,27) = 0.145; *p* = 0.866), which was indicated by similar tone-elicited freezing levels during cued fear extinction and retention on days 2 and 3, respectively. Neither the male nor female ASM−/−, ASM +/− and ASM+/+ mice showed within-extinction of cued fear (males: Figure 3b; F(19,513) = 1.356; *p* = 0.143; females: Figure 3f; F(19,513) = 1.487; *p* = 0.084) or between-extinction of cued fear (males: F(1,54) = 0.231; *p* = 0.633; females: F(1,54) = 0.260; *p* = 0.612), confirming previous studies using this paradigm in mice [46,47].

A significant genotype effect was found in the expression of contextual fear in the male mice (Figure 3d; F(2,27) = 18.743; *p* < 0.001). Both the ASM−/− and ASM+/− mice showed decreased context-elicited freezing levels compared with the ASM+/+ control mice on day 4. There was no difference in contextual fear between the male ASM−/− mice and male ASM+/− mice, suggesting that even a partial ASM deficiency is sufficient to reduce the expression of contextual fear in male mice. In the female mice, however, no such effects were found (Figure 3h). The female ASM−/−, ASM+/− and ASM+/+ mice showed similar context-elicited freezing levels on day 4 (F(2,27) = 1.012; *p* = 0.377), suggesting that ASM deficiency impairs the expression of contextual fear in male mice but not in female mice.

Altogether, these findings suggest that ASM activity modulates the expression of contextual fear but not of cued fear in a sex-specific manner, an effect that is not due to altered fear learning. An increased ASM activity leads to an increased expression of contextual fear in both male and female mice, while a decreased ASM activity leads to an impaired expression of contextual fear in male mice but not in female mice.

## 3. Discussion

Our study shows for the first time that ASM regulates contextual fear in a sex-specific manner, an effect that is not due to altered fear learning. While ASM overexpression enhances the expression of contextual fear in both male and female mice, ASM deficiency reduces the expression of contextual fear specifically in male mice. The expression of cued fear, however, is not affected by ASM overexpression or ASM deficiency.

Previous studies could show detrimental effects of increased ASM activity on anxiety- and depressive-like behavior [25,28]. We now demonstrate an enhanced expression of contextual fear in ASMtg mice, a finding that is in line with a clinical study describing increased plasma ASM activity in PTSD patients [18]. Enhanced fear responses to trauma-associated stimuli is a specific attribute of PTSD [39]. An enhanced fear expression might also be due to enhanced fear learning or to elevated fear memory. Given that the ASMtg mice showed similar fear learning compared with the WT mice, it is unlikely that the increased expression of contextual fear is due to enhanced fear learning. The question remains as to whether the enhanced expression of contextual fear might be due to elevated fear memory. The ASMtg mice showed normal social and object recognition memory [28], which are also dependent on the activity of the hippocampus. Although additional brain regions, such as the insular cortex, perirhinal cortex and medial prefrontal cortex, are necessary for the formation of recognition memory [48], it is possible that the valence of the encoded memory plays an important role. As such, an increased ASM activity might enhance the expression of aversive contextual fear memory but not of non-aversive recognition memory.

The possible mechanisms mediating the enhanced expression of contextual fear in the ASMtg mice are unclear to date but might include the effects of increased ASM and/or ceramide levels on neuropeptide Y receptor 2 (NPY2R)- or gamma-aminobutyric acid type A receptor subunit alpha5 (GABRA5)-mediated neuronal transmission. As such, the *Npy2r* and *Gabra5* genes involved in the regulation of fear responses [49,50] and known to be upregulated after exposure to contextual fear conditioning [51] were upregulated in the ASMtg mice compared with the WT mice under basal conditions [29]. An upregulation of the *Npy2r* gene was also associated with an increased expression of conditioned social fear, a process that is also dependent on the activity of the hippocampus [52,53]. Given that the NPY promoted fear extinction by acting on the NPY2R [49,54] and that the deletion of these receptors increased the expression and impaired the extinction of contextual fear [55], the upregulation of the *Npy2r* gene in the ASMtg mice might indicate a compensatory mechanism for an insufficient NPY bioavailability and, thus, NPY2R signaling. This insufficient NPY bioavailability might then lead to an increased expression of contextual fear in ASMtg mice. In support of this hypothesis, lower NPY concentrations were found in the cerebrospinal fluid of combat veterans with PTSD compared with combat veterans without PTSD [56] and healthy volunteers [57].

ASM deficiency has been previously shown to impair hippocampal-dependent memory in tests such as the Y maze [37,58] and passive avoidance test [59]. We now confirmed and extended these findings by showing that ASM deficiency also impairs a different hippocampal-dependent memory process, i.e., expression of contextual fear. The expression of cued fear, however, was not affected by ASM deficiency. As the contribution of both the hippocampus and amygdala is necessary for the expression of cued fear [60], it seems likely that ASM deficiency impairs only the hippocampal-dependent processing of information involving complex, polymodal events, such as contextual information, but not the amygdala-dependent processing of simple, modality-specific stimuli, such as a tone or light. Why such changes were not observed in females is unclear to date and needs to be investigated further, especially because the memory deficits described in the Y maze were observed in both the male and female ASM+/− mice [37,58].

In support of our results, the pharmacological inhibition of ASM activity via chronic administration of the FIASMA fluoxetine impaired the expression of contextual fear but not of cued fear in male mice [61], further supporting a specific involvement of ASM in contextual fear memory. Similarly, the chronic administration of FIASMAs, such as fluoxetine, paroxetine, imipramine and sertraline, improved symptoms of PTSD in patients and was effective in preventing symptom relapses in PTSD patients [62,63,64,65]. The question arises as to whether higher ASM activity might be a risk factor for developing PTSD in humans and vice versa, whether lower ASM activity might protect against the development of PTSD. The only study to date investigating the ASM activity in PTSD patients [18] does not permit a clear conclusion on whether the increased ASM activity is a risk factor for developing PTSD or whether this increase was the result of the disease. Although no studies to date have investigated a possible protective effect of lower ASM activity against the development of PTSD, humans carrying the single nucleotide polymorphism (SNP) rs1050239 in the *Smpd1* gene coding for ASM show lower plasma ASM activity [66] and might represent an appropriate subject group to address this question. It might also be relevant to investigate whether patients taking FIASMAs for other medical indications show attenuated contextual fear and whether they might be protected against the development of PTSD.

Given that ASM catalyzes the hydrolysis of sphingomyelin to ceramide, the mechanisms responsible for the reduced contextual fear observed in ASM deficient mice might include the effects of high sphingomyelin levels and/or of low ceramide levels on the brain, especially on the hippocampus. High sphingomyelin levels, for example, have been shown to alter the endocannabinoid system [37] and the phosphoinositide PI(4,5)P2 metabolism [59] in ASM+/− mice. Bartoll and colleagues [37] reported that high sphingomyelin levels reduced the expression of the endocannabinoid receptor CB1 in hippocampal neurons of ASM+/− mice. The CB1 receptor mediates the inhibitory and excitatory synaptic plasticity that underlies learning and memory processes [67,68]. The activation of CB_1_ receptor signaling through inhibition of the endocannabinoid-degrading enzyme fatty acid amide hydrolase (FAAH) reduced the sphingomyelin levels in the hippocampal neurons of ASM+/− mice and improved the hippocampus-dependent memory deficits in the Y maze test [37]. High sphingomyelin levels in the neurons of ASM+/− mice were also shown to reduce the synaptic phosphoinositide PI(4,5)P2 concentration and the activity of its hydrolyzing phosphatase, PLCγ [59], which plays an important role in synaptic plasticity, learning and memory [69]. The levels of membrane-bound myristoylated alanine-rich C kinase substrate (MARCKS), a protein required for PI(4,5)P2 membrane clustering and hydrolysis, was also reduced in ASM+/− mice [59]. Consequently, the intracerebroventricular infusion of a peptide that mimics the effector domain of MARCKS increased the content of PI(4,5)P2 in the synaptic membrane and improved the hippocampus-dependent memory deficits in ASM+/− mice [59]. This study suggests that the memory deficits induced by the high sphingomyelin levels in ASM+/− mice can be ameliorated by rescuing the impaired PLCγ− PI(4,5)P2-MARCKS pathway involved in synaptic plasticity. High sphingomyelin levels have also been shown to decrease the dendritic spine density and size of the hippocampal neurons of ASM+/− mice and to decrease the levels of metabotropic glutamate receptors type I (mGluR1 and mGluR5) at the synaptic membrane [58]. The pharmacological enhancement of the neutral sphingomyelinase (NSM) by the glucocorticoid dexamethasone reduced the sphingomyelin levels in the synapses of ASM+/− mice, reversed the sphingomyelin-induced dendritic spine abnormalities and improved the hippocampus-dependent memory deficits in the Y-maze test [58]. Alterations in the size and density of dendritic spines have been related to many cognitive disorders, suggesting that they form the structural basis for long-term memory [70,71]. Similar to the ASM+/− and ASM−/− mice in our study, mGluR5−/− mice show an impaired expression of contextual fear but unaltered expression of cued fear [58], suggesting that the decreased levels of mGluR5 in the hippocampal synaptic membrane of ASM deficient mice might mediate the reduced contextual fear expression.

The impaired expression of contextual fear in ASM+/− and ASM−/− mice might also be mediated by the decreased hippocampal ceramide levels observed in these mice [25]. In support of this hypothesis, diabetic mice with reduced ceramide concentration in the hippocampus and cerebellum have impaired hippocampal-dependent spatial learning and memory abilities [72]. These memory deficits could be improved by preventing ceramide depletion by administering a sphingosine-1-phosphate lyase inhibitor [72]. Given that ceramide serves as a precursor for the production of sphingosine, which can be phosphorylated by sphingosine kinases (SPHK1 and SPHK2) into sphingosine-1-phosphate, reduced ceramide levels might result in reduced sphingosine-1-phosphate levels. Reduced hippocampal sphingosine-1-phosphate levels in SphK2−/− mice were associated with deficits in spatial memory and impaired expression of contextual fear [73,74], similar to the phenotype observed in ASM deficient mice with reduced hippocampal ceramide concentration (Figure 3d, [25]).

Taken together, we have shown that ASM modulates the expression of contextual fear but not of cued fear in a sex-specific manner. Our results add a novel piece of information regarding the involvement of ASM in hippocampal-dependent aversive memory.

## 4. Materials and Methods

### 4.1. Animals

The ASMtg mice were generated by a targeted integration of a murine Smpd1 cDNA under the control of a cytomegalovirus (CMV) immediate early enhancer/chicken beta-actin fusion promoter (CAG) into the Hprt locus (Hprttm1.1(CAG-Smpd1)Jhkh) [25]. A loxP-flanked STOP cassette between the promoter and the transgene prevented constitutive overexpression. Overexpression of ASM was initiated by crossing the ASMtg female mice with homozygous E2A-Cre male mice expressing Cre recombinase under the control of an E2A promoter (Tg(EIIacre). Experiments were conducted with the ASMtg and WT littermates from the F1 generation (males: n = 13 ASMtg, n = 13 WT; females: n = 12 ASMtg, n = 12 WT). Homozygous (ASM−/−; Smpd1−/−) and heterozygous (ASM+/−; Smpd1+/−) ASM deficient mice and WT littermates (ASM+/+; 8–10 weeks old) were studied in a sex-balanced design (males: n = 10 ASM−/−, n = 11 ASM+/−, n = 9 ASM+/+; females: n = 10 ASM−/−, n = 11 ASM+/−, n = 9 ASM+/+). Mice were held under standard laboratory conditions (12:12 light:dark cycle, lights on at 07:00 h, 22 °C, 60% humidity, food and water ad libitum). Experiments were performed during the light phase between 09:00 and 14:00 in accordance with the Guide for the Care and Use of Laboratory Animals of the Government of Unterfranken (project identification code 55.2-2532.1-27/11 approved on 7 September 2015) and the guidelines of the NIH.

### 4.2. Fear Conditioning Apparatus

The conditioned fear experiments were performed in two different contexts, A and B, which differed in visual, tactile and olfactory cues as previously described [46,47] (Figure 1). Briefly, fear conditioning and contextual fear measurement occurred in context A, which consisted of a transparent Perspex box (23 × 23 × 36 cm) with an electric grid floor. Context A was cleaned with water containing a small amount of a neutral smelling detergent before each trial. Cued fear extinction training and retention occurred in a different context (context B) to allow the measurement of cued fear responses independently from the confounding influence of contextual memory. Context B consisted of a black Perspex box (23 × 23 × 36 cm) with a smooth floor and was cleaned with water containing a small amount of a lemon-scented detergent before each trial. The boxes were enclosed in a wooden chamber to reduce external noise and visual stimulation. A low level of background noise was produced by ventilation fans within the chamber. Illumination (300 lx for context A and 20 lx for context B) was provided by four white light-emitting diodes. Auditory stimuli were delivered through a speaker attached 30 cm above the floor of the box. Freezing, defined as the absence of all movement except that required for respiration [40], was measured with the computerized fear conditioning system (TSE System GmbH, Bad Homburg, Germany). The conditioning chamber contained two horizontal detection fields, each with 16 infrared light beams set 1.3 cm apart. Inactivity was measured by the infrared beams and defined as no light beam interruption for at least 1 s.

### 4.3. Fear Conditioning Procedure

#### 4.3.1. Fear Conditioning (Day 1)

The mice were placed in the conditioning chamber (context A) and, after a 5-min adaptation period, were exposed to five CS–US pairings with a 2 min inter-stimulus interval. The CS was an 80 dB, 8 kHz, 30 s sine-wave tone, which co-terminated with a mild electric foot shock (US, 0.7 mA; pulsed current, 2 s). The mice were returned to their home cage 5 min after the last CS–US pairing.

#### 4.3.2. Cued Fear Extinction Training (Day 2)

One day after fear conditioning, the mice were placed in context B and, after a 5-min adaptation period, exposed to 20 CS presentations (30 s sine-wave tone, 5 s inter-stimulus interval). The mice were returned to their home cage 5 min after the last CS presentation.

#### 4.3.3. Cued Fear Extinction Retention (Day 3)

One day after cued fear extinction training, the mice were again placed in context B; after a 5-min adaptation period, they were exposed to five CS presentations (30 s sine-wave tone, 5 s inter-stimulus interval). The mice were returned to their home cage after the last CS presentation.

#### 4.3.4. Contextual Fear Measurement (Day 4)

Contextual fear was tested one day later by measuring freezing behavior during a 5 min exposure to the original conditioning context A. These 5 min were shown as 10 × 30 s time-bins.

### 4.4. Statistical Analysis

For the statistical analysis, SPSS (Version 28, SPSS Inc., Chicago, IL, USA) was used. Data were analyzed by two-way ANOVA for repeated measures, followed by Bonferroni’s post hoc analysis whenever appropriate. Statistical significance was set at *p* < 0.05.

## Data Availability

The datasets generated during the current study are available from the corresponding author on request.
